# Hypoxia-inducible transcription factor-1α inhibition by topotecan protects against lipopolysaccharide-induced inflammation and apoptosis of cardiomyocytes

**DOI:** 10.1186/s12938-021-00923-2

**Published:** 2021-08-31

**Authors:** Ying Zhang, Yi Xu, Ke Zhou, Guoying Kao, Meng Yan, Jun Xiao

**Affiliations:** 1 Department of Cardiovascular Medicine, Chongqing University Center Hospital (Chongqing Emergence Medical Center), No. 1, Jiankang Road, Yuzhong District, Chongqing, 400014 China; 2 Department of Nursing, Chongqing Gaoxin District People’s Hospital, Chongqing, 400039 China

**Keywords:** Myocarditis, Inflammation, Apoptosis, HIF-1α, Topotecan

## Abstract

**Background:**

Myocarditis, an inflammatory disease of the myocardium, is a serious hazard to human life due to the expansion of inflammatory lesions in the myocardium. The aim of this study was to investigate the role of hypoxia-inducible transcription factor (HIF)-1α and its inhibitor topotecan in the pathogenesis of myocarditis.

**Methods:**

H9c2 cardiomyoblasts was stimulated with lipopolysaccharide (LPS) to simulate myocarditis model in vitro. The levels of myocardial damage markers were determined using commercially available kits. Western blotting was used to evaluate HIF-1α expression after LPS challenge. Then, after HIF-1α silencing, the contents of inflammatory factors were determined with enzyme-linked immunosorbent assay (ELISA). Cell viability was tested by means of a cell counting kit-8 (CCK-8) assay. Cell apoptosis was assessed by flow cytometry, and the expression of apoptotic proteins was examined using western blot analysis. Subsequently, HIF-1α was overexpressed and topotecan was employed to treat H9c2 cells under LPS exposure condition. The biological functions were detected again.

**Results:**

LPS significantly elevated the levels of lactate dehydrogenase (LDH), creatine kinase-MB (CK-MB) and cardiac troponin-I (cTn-I) in supernatant of H9c2 cell lysates. Additionally, LPS led to the notably upregulated expression of HIF-1α. HIF-1α-knockdown markedly decreased the concentrations of tumor necrosis factor (TNF)-α, interleukin (IL)-6 and IL-8 compared with the LPS-induced group. Moreover, the cell viability was conspicuously enhanced and cell apoptotic ratio was remarkably reduced, accompanied by downregulated expression of Bax, Bim, caspase 3 and caspase 9 after HIF-1α silencing. Consistently, HIF-1α gain-of-function significantly promoted the production of inflammatory cytokines and cell apoptosis, which was partially counteracted by topotecan administration.

**Conclusion:**

To conclude, these findings demonstrated that HIF-1α inhibition by topotecan ameliorates LPS-induced myocarditis in vitro, providing a new approach in the treatment of myocarditis.

## Background

Myocarditis, also known as inflammatory cardiomyopathy, is a disease featured by the existence of inflammatory infiltrates in myocardial tissue in the clinic and histology with a wide range of symptoms in children and young adults [1]. Persistent myocarditis potentially contributes to the structural and functional abnormalities in cardiomyocytes, which is the leading cause of acute cardiac failure, dilated cardiomyopathy and sudden cardiac death [2]. Therefore, elucidating the detailed molecular mechanism underlying the progression of myocarditis will provide an effective strategy for the treatment of this disease.

Myocarditis is mainly induced by infectious and noninfectious factors, including bacterial infection, viral infection, drug reaction and autoimmune disease [3]. With the increase in immune-compromised hosts, the number of patients with bacterial myocarditis has recently been increasing [4]. Lipopolysaccharide (LPS; also known as endotoxin), the chief component of the cell wall of Gram-negative bacteria, is one of the key mediators of inflammation, which often triggers systemic inflammation, multiple organ failure, heart involvement and cardiac function damage. Therefore, LPS is widely used to simulate myocardial cytotoxicity in a large number of studies on the pathogenesis of myocarditis [5, 6].

Hypoxia-inducible transcription factor (HIF)-1 is a heterodimeric transcription factor whose expression is regulated by oxidative stress and inflammatory cytokines [7]. The stability of HIF-1α subunit is regulated by the family of oxygen-dependent hydrolases. It has been reported that the expression of HIF-1α in cardiac leukocytes correlates with the severity of myocarditis in end-stage Chagas disease patients [8]. Topotecan, an inhibitors of HIF-1α, has been demonstrated to mitigate LPS-induced acute lung injury by the NF-kappa B signaling [9]. By reducing inflammation, topotecan can alleviate the development of radiation necrosis in the mouse brain by inhibiting HIF-1α expression [10]. These findings have aroused strong interest to explore the effect of HIF-1α on the pathogenesis of myocarditis.

In the present study, we aimed to explore the roles of HIF-1α and topotecan in the inflammation and apoptosis of cardiomyocytes induced by LPS, which is an in vitro cell model to simulate myocarditis followed by Gram-negative bacterial infection. Findings in this study may provide a new approach in the treatment of myocarditis.

## Results

### LPS exposure significantly elevates the levels of myocardial damage markers

**Fig. 1:**
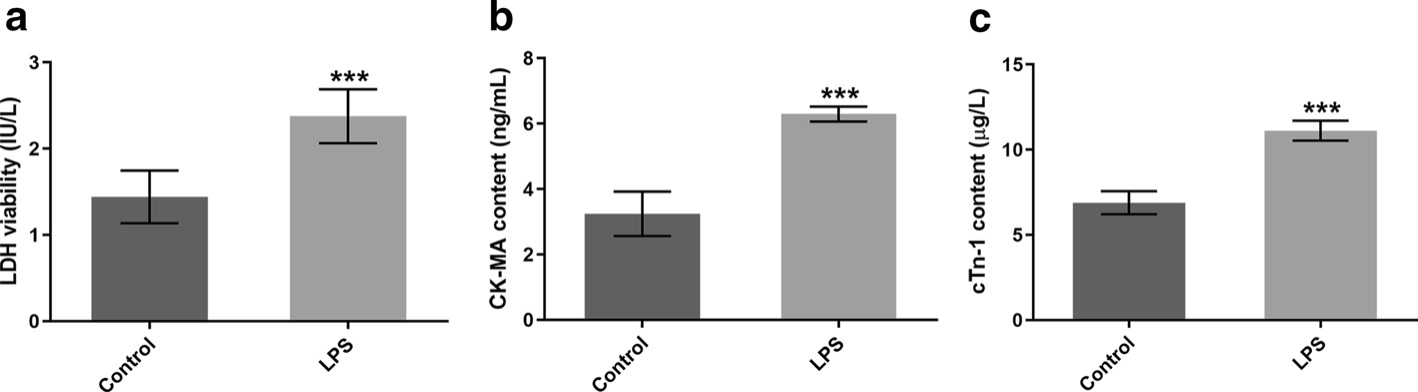
LPS exposure significantly increased the contents of myocardial damage markers in H9c2 cells. The concentrations of **A** LDH, **B** CK-MB and **C** cTn-1 were detected using the corresponding commercial available kits. ^***^P < 0.001 vs. control

Firstly, to assess the establishment of myocarditis model in vitro, the concentrations of markers reflecting myocardial injury including LDH, CK-MB and cTn-1 were determined using the corresponding kits. As shown in Fig. 1A–C, LPS challenge markedly enhanced the levels of LDH, CK-MB, cTn-1 in comparison to the control group, suggesting the successful induction of the cardiomyocytes injury model.

### HIF-1α is highly expressed in LPS-stimulated H9c2 cells

**Fig. 2:**
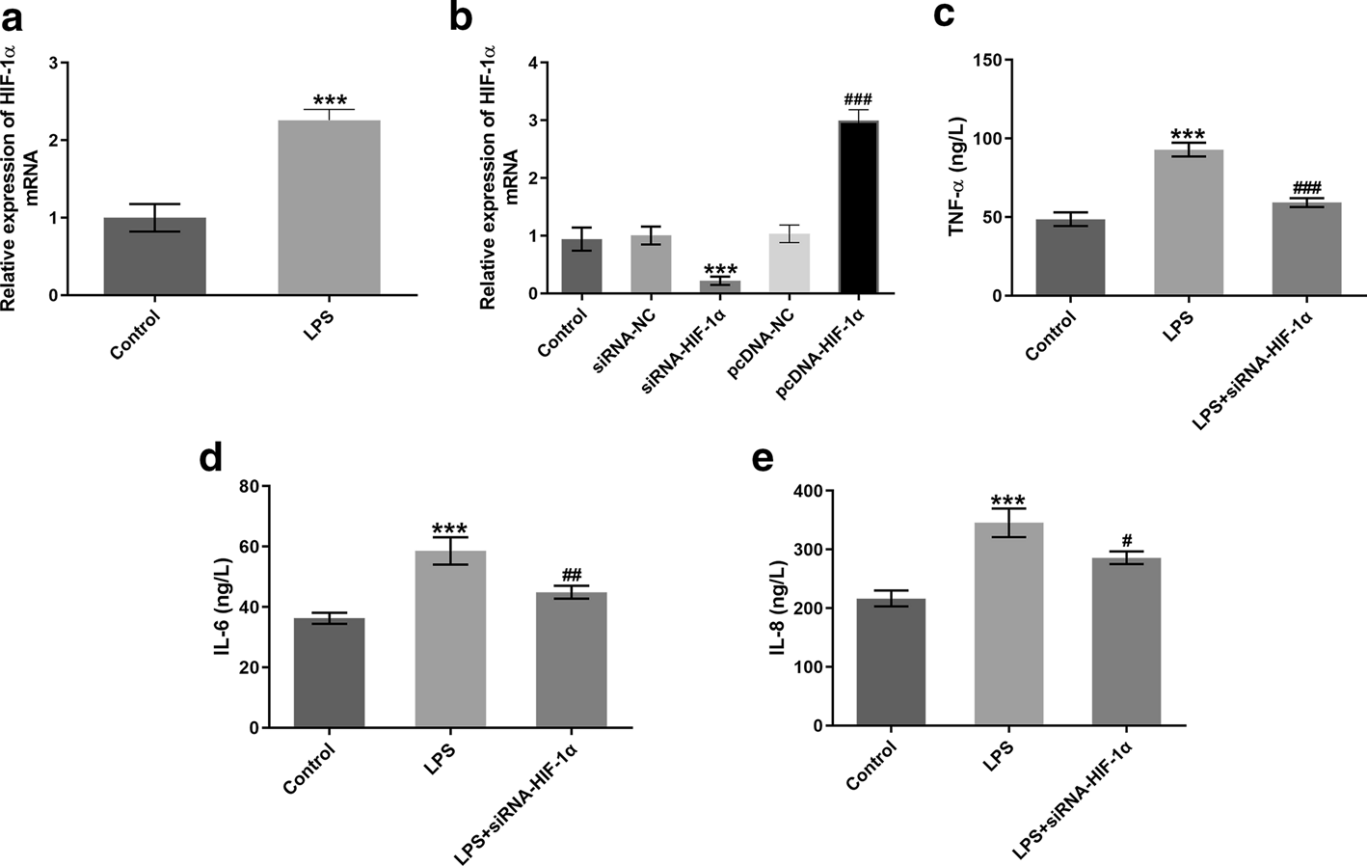
HIF-1α expression was markedly upregulated in LPS-stimulated H9c2 cells, and HIF-1α ablation ameliorated LPS-induced inflammatory responses of cardiomyocytes H9c2 cells. **A** The expression of HIF-1α was determined with RT-qPCR after LPS exposure. ^***^P < 0.001 vs. control. **B** RT-qPCR was used to evaluate the level of HIF-1α following transfection. ^***^P < 0.001 vs. siRNA-NC; ^###^P < 0.001 vs. pcDNA-NC. **C**–**E** The secretion levels of TNF‐α, IL‐6 and IL‐8 were tested by means of ELISA kits. ^***^P < 0.001 vs. control; ^#^P < 0.05, ^##^P < 0.01, ^###^P < 0.001 vs. LPS

Subsequently, the expression of HIF-1α in H9c2 cells exposed to LPS was examined by means of RT-qPCR. As observable from Fig. 2A, HIF-1α expression was remarkably upregulated in the LPS challenge group compared with the control group. Afterwards, HIF-1α expression was silenced or overexpressed by transfection with siRNA-HIF-1α or pcDNA-HIF-1α. It was found that cells transfected with siRNA-HIF-1α displayed reduced HIF-1α level relative to the control group (Fig. 2B). By contrast, notably increased HIF-1α level was observed following pcDNA-HIF-1α transfection when compared to the empty vector group.

### HIF-1α silencing alleviates LPS-induced inflammatory injury of cardiomyocytes H9c2 cells

We further study the effects of HIF-1α depletion on the regulation of inflammatory responses following LPS exposure. Results presented in Fig. 2C–E indicated that LPS induction triggered conspicuously intensified levels of TNF‐α, IL‐6 and IL‐8 in comparison to the control group. On the contrary, in comparison with H9c2 cells treated with LPS alone, HIF-1α silencing dramatically reduced the concentrations of above-mentioned inflammatory factors. These observations reveal that HIF-1α silencing attenuates inflammatory responses induced by LPS in cardiomyocytes H9c2 cells.

### HIF-1α silencing inhibits cell apoptosis in LPS-injured cardiomyocytes H9c2 cells

**Fig. 3:**
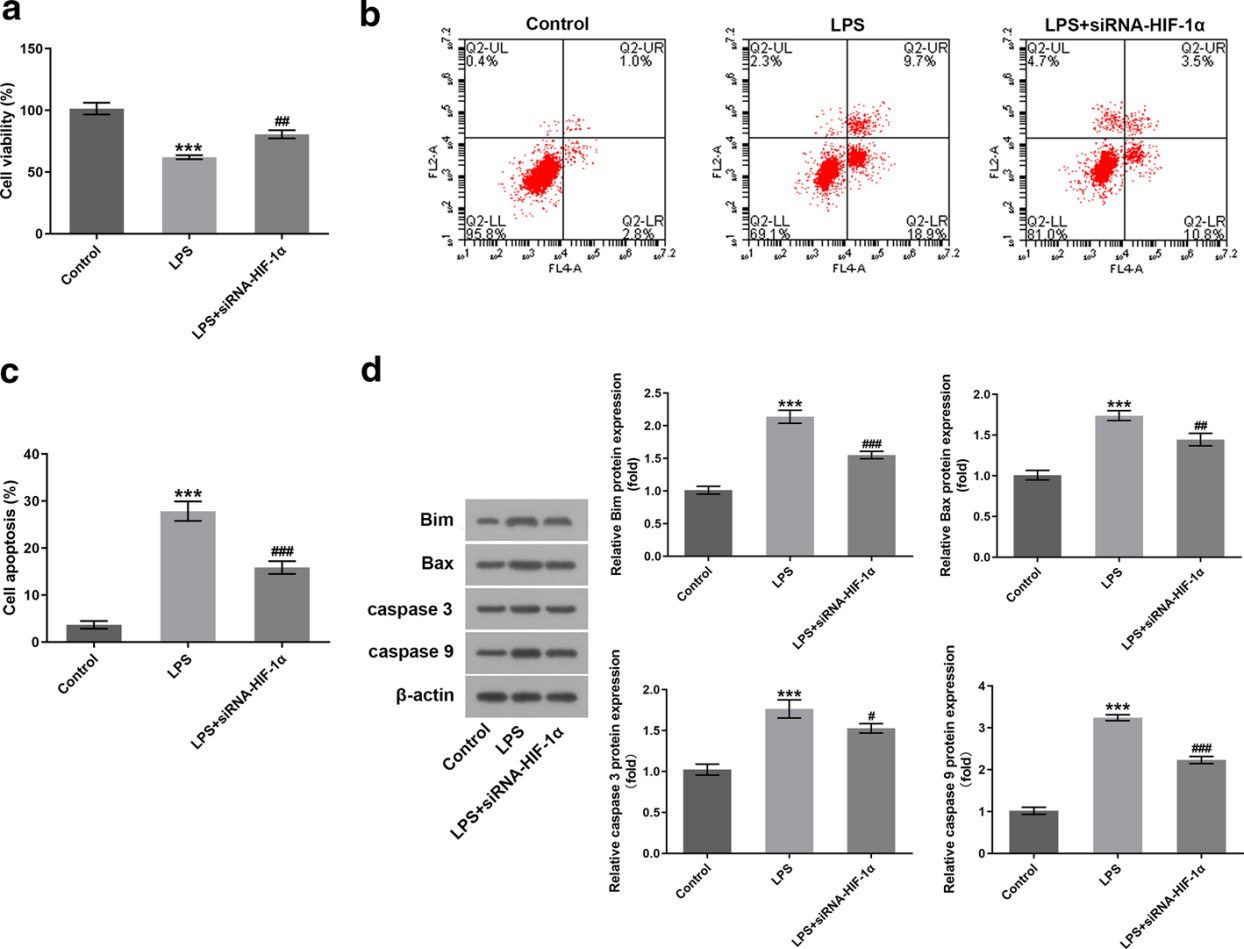
HIF-1α silencing inhibited cell apoptosis in LPS-injured cardiomyocytes H9c2 cells. **A** Cell viability was examined by a CCK-8 assay. **B**, **C** Cell apoptosis ratio was determined with flow cytometry. **D** Western blotting was exploited for assessing the expression of apoptosis-related proteins. ^***^P < 0.001 vs. control; ^#^P < 0.05, ^##^P < 0.01, ^###^P < 0.001 vs. LPS

To explore the biological function of HIF-1α silencing on LPS‐stimulated H9c2 cell injury, cell viability was evaluated using a CCK-8 assay. As exhibited in Fig. 3A, LPS induction significantly decreased cell viability relative to the control group, while HIF-1α-knockdown partially counteracted the inhibitory effect of LPS on cell viability. Then, cell apoptosis was tested by flow cytometry. Results from Fig. 3B, C revealed that LPS exposure elevated cell apoptosis rate compared with the control group. On the contrary, inhibition of HIF-1α reduced the ratio of H9c2 cell apoptosis-induced by LPS.

Consistently, markedly upregulated expression of Bax, Bim, caspase 3 and caspase 9 was noticed following LPS stimulation, which was reversed after HIF-1α depletion (Fig. 3D). Through the above findings, we prove that HIF-1α deficiency suppresses cell apoptosis in LPS-injured cardiomyocytes H9c2 cells.

### Topotecan intervention abrogates the impact of HIF-1α-upregulation on the inflammation and apoptosis in LPS-induced H9c2 cells

**Fig. 4:**
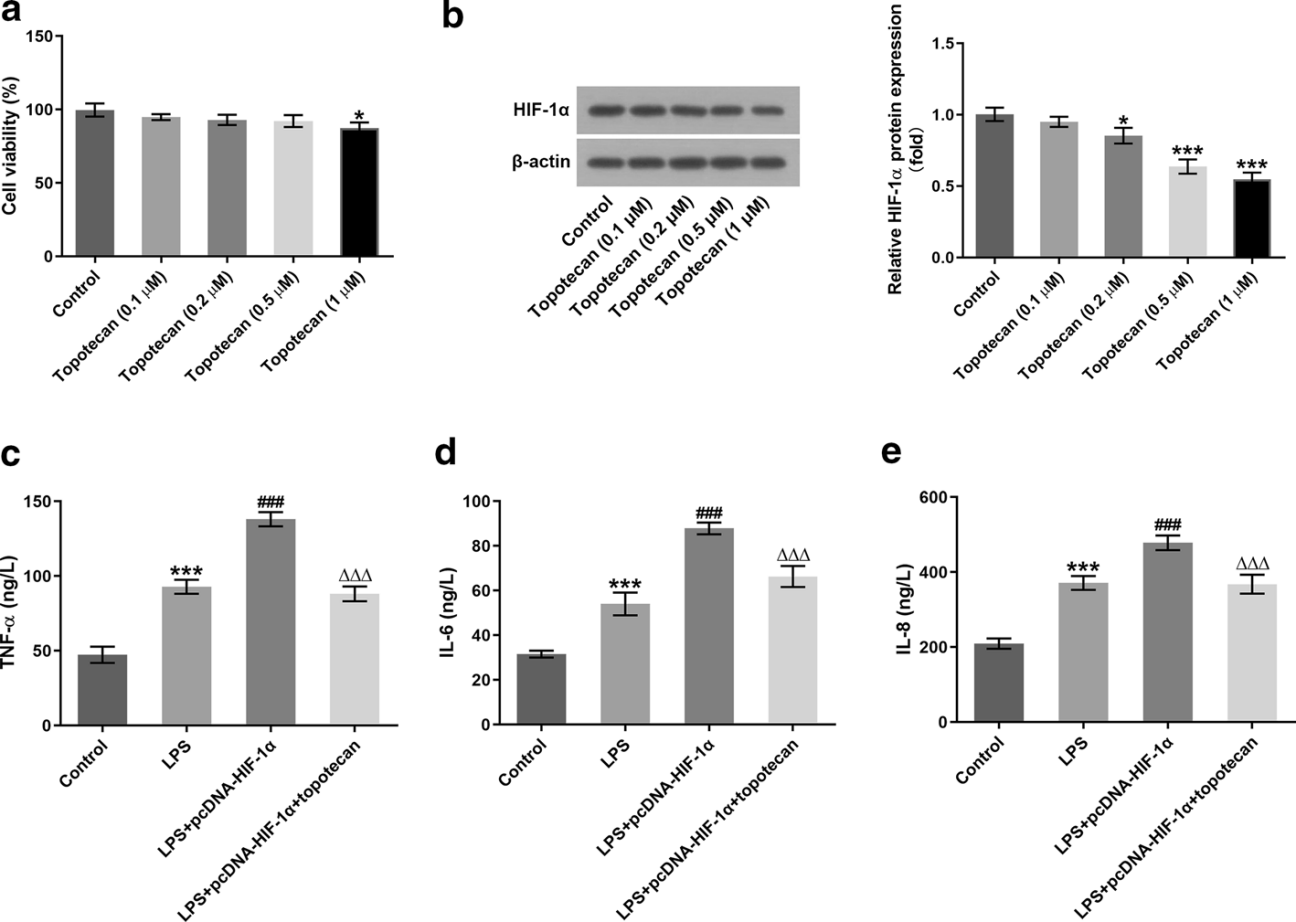
Topotecan intervention abrogated the impact of HIF-1α-upregulation on the inflammation in LPS-induced H9c2 cells. **A** Cell viability was measured using a CCK-8 assay after topotecan treatment. **B** The expression of HIF-1α was tested by means of western blot analysis after topotecan intervention. ^*^P < 0.05, ^**^P < 0.01, ^***^P < 0.001 vs. control. **C**–**E** ELISA kits were used to determine the levels of TNF-α, IL-6 and IL-8. ^***^P < 0.001 vs. control; ^###^P < 0.001 vs. LPS; ^△△△^P < 0.001 vs. LPS + pcDNA-HIF-1α

**Fig. 5:**
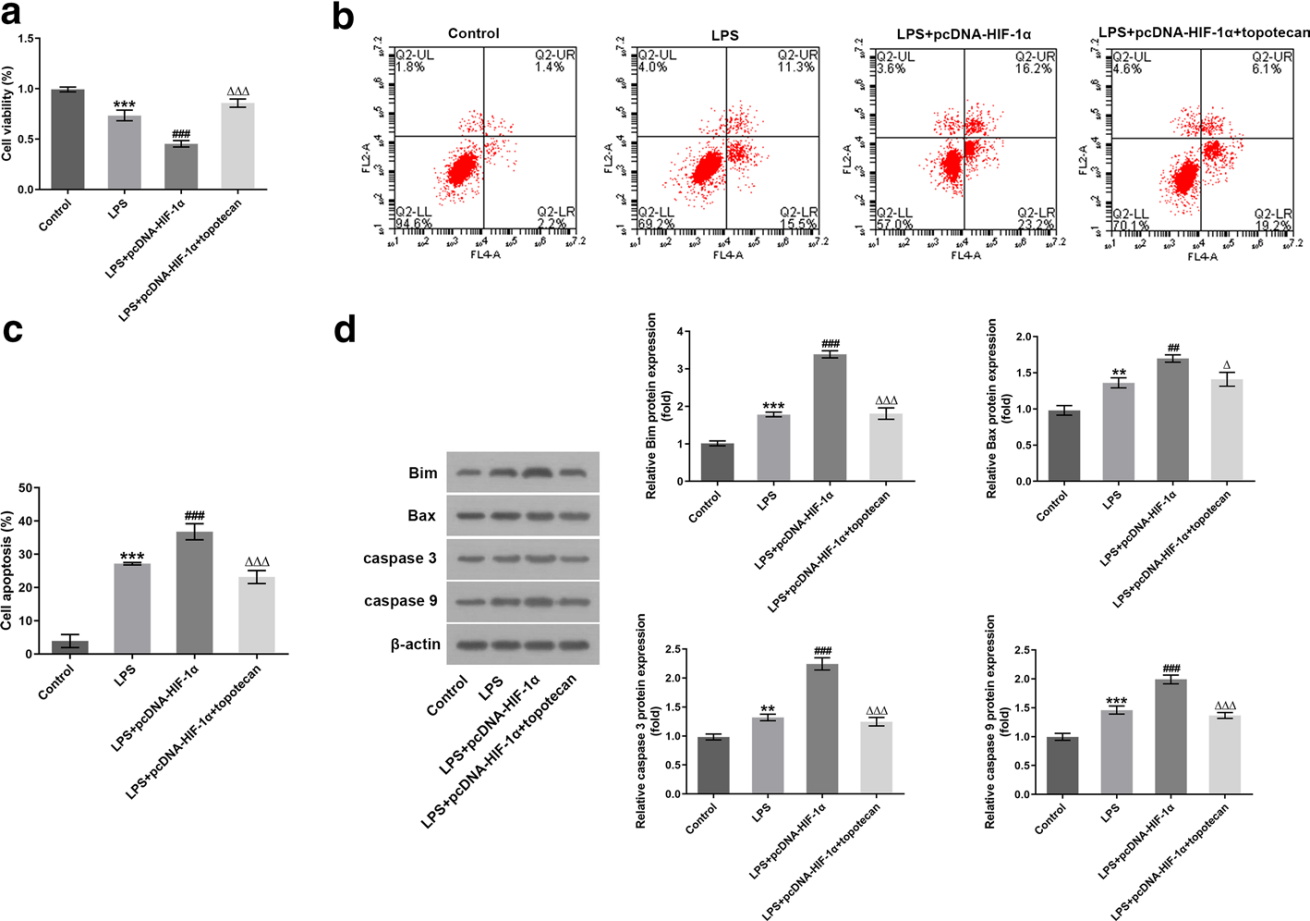
Topotecan treatment blocked the inhibitory effects of HIF-1α overexpression on the apoptosis in LPS-induced H9c2 cells. **A** Cell viability was detected using a CCK-8 assay. **B**, **C** Cell apoptosis ratio was evaluated with flow cytometry. **D** Western blotting was employed to examine the expression of apoptosis-related proteins. ^**^P < 0.01, ^***^P < 0.001 vs. control; ^##^P < 0.01, ^###^P < 0.001 vs. LPS; ^△^P < 0.05, ^△△△^P < 0.001 vs. LPS + pcDNA-HIF-1α

To exactly evaluate the role of HIF-1α in LPS-stimulated H9c2 cells, topotecan, an inhibitor of HIF-1α, was executed for treating cells. As presented in Fig. 4A, topotecan at the concentrations of 0.1, 0.2 and 0.5 µM had no significant difference on cell viability compared with the control group. However, cells exposed to 1 µM topotecan displayed marked decrease in cell viability. Additionally, topotecan dose-dependently downregulated the expression of HIF-1α in comparison to the control group (Fig. 4B). According to above findings, 0.5 µM topotecan was employed to perform the following experiments. As observable from Fig. 4C–E, HIF-1α overexpression apparently enhanced the contents of TNF‐α, IL‐6 and IL‐8 in comparison to the LPS-treated group, which were restored by topotecan intervention. Additionally, notably decreased H9c2 cell viability was found following HIF-1α overexpression, whereas topotecan restored the inhibitory effect of HIF-1α overexpression on cell viability (Fig. 5A). Moreover, it was observed that HIF-1α upregulation conspicuously elevated the apoptotic ratio compared with H9c2 cells treated with LPS alone, which was partially counteracted by topotecan administration (Fig. 5B, C). Besides, western blot analysis indicated that LPS promoted Bax, Bim, caspase 3 and caspase 9 expression, and gain‐of‐function of HIF-1α further upregulated the expression of above-mentioned proteins after exposure of H9c2 cells to LPS (Fig. 5D). By contrast, topotecan intervention mitigated the impact of HIF-1α upregulation on the level of apoptotic proteins. Together, the data of present study provide evidence that topotecan intervention blocks the impact of HIF-1α-upregulation on the inflammation and apoptosis in LPS-induced H9c2 cells.

## Discussion

To our knowledge, myocarditis is a vital cause of cardiovascular disease morbidity and death in children and young adults [11]. Therefore, there is an urgent requirement to have a better understanding of the molecular mechanism underlying the development of myocarditis to discover an efficient target for the treatment of myocarditis. In the present study, LPS was used to induce myocardial injury in H9c2 cardiomyocytes to simulate a model of myocarditis in vitro. We demonstrated that HIF-1α was overexpressed in H9c2 cell exposed to LPS, and inhibition by topotecan could protect against LPS-induced inflammation and apoptosis in H9c2 cells.

A number of clinical trials revealed that elevated levels of TNF-α, IL-6, IL-8 and other pro-inflammatory cytokines participated in the pathogenesis of cardiac damage during myocarditis [12, 13]. Excessive apoptosis of cardiomyocytes was observed in myocarditis model in vivo and in vitro [14, 15]. Therefore, the regulation of inflammatory responses and apoptosis is considered to be an underlying treatment strategy for myocarditis. LPS is widely used to mimic myocardial cytotoxicity in a large number of studies on the pathogenesis of myocarditis [16, 17]. Similarly, in this study, we found that LPS induction caused remarkable increase in production of inflammatory cytokines TNF-α, IL-6 and IL-8 in H9c2 cells and promotion in cell apoptosis. Existing study has shown that HIF-1α expression in cardiac leukocytes correlates with the severity of myocarditis in end‐stage Chagas disease patients [8]. It is worthy of note that HIF-1α is closely associated with disease severity in knee osteoarthritis [18]. HIF-1α is significantly increased in polyp tissues, which promotes neutrophilic inflammation in chronic rhinosinusitis with nasal polyps patients [19]. Importantly, agent that inhibits HIF-1α-induced inflammation and apoptosis in macrophages can be used for the treatment of atherosclerosis [20]. HIF-1α silence dramatically reduces apoptosis and inflammation in H9c2 cell during high glucose stress [21]. Consistent with the above research, this study suggested that HIF-1α deficiency apparently restrains LPS-induced inflammation and apoptosis in H9c2 cells.

To further clarify the role of HIF-1α in LPS-stimulated H9c2 cells, HIF-1α was overexpressed by transfection with pcDNA-HIF-1α, and topotecan, an inhibitor of HIF-1α, was executed for treat cells under LPS exposure condition. Topotecan is reported to attenuate LPS-mediated acute lung injury via the NF-κB pathway [22]. Additionally, Oleg et al. [23] demonstrated that targeted inhibition of HIF-1 might represent a novel neuroprotective strategy by preventing neuronal apoptosis in rat hippocampus caused by severe hypoxia. Compelling evidence indicates that topotecan administration inhibits HIF-1a expression and improves retinal ganglion cell survival leading to a functional protection against retinal ischemia–reperfusion [24]. The present study indicated that topotecan intervention abrogated the impact of HIF-1α upregulation on the inflammation and apoptosis in LPS-induced H9c2 cells.

## Conclusions

To summarize briefly, findings gained in the present study for the first time corroborate that HIF-1α participates in the development of myocarditis, and HIF-1α silencing markedly inhibits LPS-triggered inflammation and apoptosis of H9c2 cells, while HIF-1α overexpression plays the opposite role. Importantly, topotecan intervention partially counteracts the impact of HIF-1α-upregulation on the inflammation and apoptosis in LPS-induced H9c2 cells. These findings provide a new understanding on the mechanism underlying myocarditis and a new approach in the treatment of myocarditis. However, the lack of study about the effects of HIF-1α and topotecan in myocarditis animal model is a limitation of the present research and therefore, a comprehensive analysis is required in the future.

## Materials and methods

### Cell culture and treatment

H9c2 cardiomyoblasts were obtained from The Cell Bank of Type Culture Collection of Chinese Academy of Sciences (Shanghai, China). Cells were cultured in Dulbecco’s Modified Eagle medium (DMEM; Gibco, Grand Island, USA) containing 10% fetal bovine serum (FBS, Gibco, Grand Island, USA). The incubator was set as 5% CO_2_ humidified atmosphere at 37 °C. H9c2 cells were dealt with 10 μg/ml of LPS (Sigma-Aldrich, MO, USA) for 12 h to construct the cell injury model of myocarditis, which was according to the previous study [25]. Various gradient concentrations (0.1, 0.2, 0.5 and 1 µM) of topotecan were used to pretreat cells for 6 h before LPS exposure.

### Cell transfection

H9c2 cells in logarithmic phase were collected and loaded into 6-well plates (1 × 10^6^ per well), which were then incubated at 37 °C until 80% confluence. Small interfering RNA RNA (siRNA) directed against HIF-1α (siRNA-HIF-1α) and HIF-1α overexpressed plasmid (pcDNA-HIF-1α) were designed and synthesized by GenePharma Corporation (Shanghai, China). The transfection procedure was carried out using Lipofectamine 2000 Reagent (Invitrogen, Carlsbad, CA, USA) according to the manufacturer’s protocol. Cells were harvested 48 h following transfection and the transfection efficiency was determined using reverse transcription-quantitative PCR (RT-qPCR).

### Cell viability assay

H9c2 cells were seeded in a 96-well tissue culture plate with 5000 cells/well, and cell viability was determined with Cell Counting Kit-8 (CCK-8; Shanghai Yi Sheng Biotechnology Co. Ltd., Shanghai, China). In brief, when the treatments were completed, 10 µl CCK-8 solution was added to each well, and the cultures were incubated at 37 °C for a further 2 h. The optical density (OD) at 450 nm of each sample was measured utilizing a microplate reader (Bio-Rad, Hercules, CA).

### Detection of myocardial markers

The cells were lysed by RIPA lysis buffer (Beyotime, Shanghai, China) and culture supernatant of cell lysates was obtained by centrifugation at 4 °C (1000 g for 10 min). The expression levels of lactate dehydrogenase (LDH), creatine kinase-MB (CK-MB) and cardiac troponin-I (cTn-I) were evaluated by means of the corresponding commercial available kits (Nanjing Jiancheng Bioengineering Institute, Nanjing, china) following manufacturer's recommendations.

### Test for the concentrations of inflammatory factors

The levels of inflammatory factors including tumor necrosis factor (TNF)-α, interleukin (IL)-6 and IL-8 in culture supernatant were tested using enzyme-linked immunosorbent assay (ELISA) according to protocols supplied on the basis of user’s manual delivered by the producer (Shanghai Xitang Biological Technology Co., Ltd., Shanghai, China).

### Apoptosis assay

After cells had been subjected to the treatments as described above, cells were washed twice gently with cold phosphate buffered saline (PBS). The apoptosis ratios of H9c2 cells were conducted using fluorescein isothiocynate (FITC)-conjugated Annexin V and propidium iodide (PI) staining method (Invitrogen, Carlsbad, CA, USA) on a FACScan flow cytometry (Beckman Coulter, Atlanta, GA, USA). The data were analyzed by using FlowJo software (TreeStar, Ashland, OR, USA).

### Quantitative reverse transcription polymerase chain reaction (RT-qPCR) analysis

Total RNA was extracted from H9c2 cells using TRIzol reagent (Invitrogen; Thermo Fisher Scientific, Inc.) in accordance with the specification provided by the supplier. Then, complementary DNA (cDNA) was synthesized using the PrimeScript RT Reagent Kit (Takara, Japan). qPCR was then performed with 2 μg cDNA as the templet by Power SYBR Master Mix (Applied Biosystems, Foster, USA) on the ABI 7500 PCR system (Applied Biosystems). GAPDH was used as the internal reference gene. The 2^−ΔΔCt^ method was utilized for the quantification of targeted mRNA.

### Western blot analysis

Cells were harvested after treatment and the RIPA lysis buffer (Beyotime Institute of Biotechnology) was used to obtain the cellular lysate samples. After quantification using a bicinchoninic acid protein assay kit (Beyotime Institute of Biotechnology), the protein lysate samples (40 µg/lane) was separated in a 10% SDS-polyacrylamide gel by electrophoresis and then transferred onto polyvinylidene difluoride (PVDF; Millipore, Billerica, MA, USA) membrane. Subsequently, these membranes were blocked with 5% non-fat milk at room temperature for 1.5 h. After incubation with specific primary antibodies (Abcam Company, Cambridge, UK) at 4 °C overnight and secondary antibody conjugated with horseradish peroxidase (Cell Signaling Technology, Boston, MA, USA) at room temperature for 1 h, protein bands were visualized with enhanced chemiluminescence substrate (Pierce, USA) using chemiluminescence imaging equipment (Claremont, CA, USA). The intensity of the bands was quantified by using Image J software (version 1.45; National Institutes of Health). β-actin served as the internal control.

### Statistical analysis

The measurement data were described as the mean ± standard deviation. Three biological replications were conducted for each experiment. Data management and analysis were performed by GraphPad Prism version 8.0 (GraphPad Software, Inc.). Comparisons between two groups were performed using Student’s *t*-test. One-way analysis of variance (ANOVA) with Tukey's post hoc test was used to conduct to assess multiple differences. A *P* value < 0.05 was considered statistically significant.

## Data Availability

The raw data supporting the conclusions of this article are available from the corresponding author on reasonable request.
